# Association Between Wait Time of Central Venous Pressure Measurement and Outcomes in Critical Patients With Acute Kidney Injury: A Retrospective Cohort Study

**DOI:** 10.3389/fpubh.2022.893683

**Published:** 2022-08-09

**Authors:** Qilin Yang, Weixiao Chen, Yichao Wen, Jiezhao Zheng, Jieru Chen, Shuilian Yu, Xiaohua Chen, Weiyan Chen, Xuming Xiong, Deliang Wen, Zhenhui Zhang

**Affiliations:** ^1^Department of Critical Care, The Second Affiliated Hospital of Guangzhou Medical University, Guangzhou, China; ^2^Department of Rheumatology, The Second Affiliated Hospital of Guangzhou Medical University, Guangzhou, China

**Keywords:** wait time, central venous pressure, acute kidney injury, in-hospital mortality, MIMIC-IV

## Abstract

**Background:**

Hemodynamic management is of paramount importance in patients with acute kidney injury (AKI). Central venous pressure (CVP) has been used to assess volume status. We intended to identify the optimal time window in which to obtain CVP to avoid the incidence of adverse outcomes in patients with AKI.

**Methods:**

The study was based on the Medical Information Mart for Intensive Care (MIMIC) IV database. The primary outcome was in-hospital mortality. Secondary outcomes included the number of ICU-free days and norepinephrine-free days at 28 days after ICU admission, and total fluid input and fluid balance during the first and second day. A time–dose–response relationship between wait time of CVP measurement and in-hospital mortality was implemented to find an inflection point for grouping, followed by propensity-score matching (PSM), which was used to compare the outcomes between the two groups.

**Results:**

Twenty Nine Thousand and Three Hundred Thirty Six patients with AKI were enrolled, and the risk of in-hospital mortality increased when the CVP acquisition time was >9 h in the Cox proportional hazards regression model. Compared with 8,071 patients (27.5%) who underwent CVP measurement within 9 h and were assigned to the early group, 21,265 patients (72.5%) who delayed or did not monitor CVP had a significantly higher in-hospital mortality in univariate and multivariate Cox regression analyses. After adjusting for potential confounders by PSM and adjusting for propensity score, pairwise algorithmic, overlap weight, and doubly robust analysis, the results were still stable. The HRs were 0.58–0.72, all *p* < 0.001. *E*-value analysis suggested robustness to unmeasured confounding.

**Conclusions:**

Among adults with AKI in ICU, increased CVP wait time was associated with a greater risk of in-hospital mortality. In addition, early CVP monitoring perhaps contributed to shortening the length of ICU stays and days of norepinephrine use, as well as better fluid management.

## Introduction

Acute kidney injury (AKI) is a broad clinical syndrome defined by an abrupt decrease in kidney function that includes, but is not limited to, acute renal failure. AKI has been considered to be a major public health problem affecting millions of people all over the world, leading to reduced survival, progression of underlying chronic kidney disease (CKD), and an occasional onset of new CKD (1). Fluid therapy is important for renal recovery after AKI (2). Fluid insufficiency or overload are all associated with an increased risk for mortality in critically ill patients with AKI (3).

Central venous pressure (CVP) monitoring remains the most frequently used indicator for guiding fluid resuscitation in critically ill patients (4, 5). In particular, the risk of fluid-overload-related peripheral edema, ascites, and renal impairment are associated with the absolute CVP value (6, 7). However, CVP is influenced by many factors, such as thoracic, pericardial, and abdominal pressures (5). CVP monitoring has been challenged in many studies and meta-analyses, which have reported that there was no evidence to support the use of CVP to guide fluid therapy (8, 9). Conversely, several studies have indicated that extreme CVP values could help to predict fluid responsiveness (10, 11). The overall effect of monitoring of CVP on patients with AKI is still unknown, and recommendations on when an initial CVP should be obtained are also limited. Therefore, we conducted a retrospective cohort study to determine the association between wait time of CVP initiation and outcomes in critical patients with AKI.

## Methods

### Data Sources and Setting

A population-based cohort study was conducted using critical care databases in Medical Information Mart for Intensive Care (MIMIC)-IV (version 1.0), which was built upon the MIMIC-III database (12, 13). MIMIC-IV included 76,540 ICU stays between 2008 and 2019. Yang Q obtained approval to access this database (certification number 7634793). The data has been previously de-identified, and the institutional review boards of the Massachusetts Institute of Technology (No. 0403000206) and Beth Israel Deaconess Medical Center (2001-P-001699/14) both approved the use of the database for research. We have also complied with all relevant ethical regulations regarding the use of the data for our study.

### Study Population

Adults (older than 18 years) who fulfilled the definition of AKI within 7 days after ICU admission in MIMIC-IV were eligible for our study. AKI was defined according to the Kidney Disease Improving Global Outcomes (KDIGO) criteria as previously reported (14, 15). Even though some patients were recurrently admitted to ICU, we considered only the first hospital and first ICU admission. Data of patients on second or more ICU admissions would be excluded.

### Main Exposure

The primary independent variable was the wait time for CVP, which is defined as the total time elapsed from ICU admission until initial CVP measurement (in hours). Only the first CVP value of each patient was used in this study.

### Covariates

Patient characteristics that were previously shown to explain most of the variation in mortality after AKI were measured. The following variables were included in our study: registered information of admission (age, sex, admission year), vital signs, and laboratory tests (heart rate, mean arterial pressure (MAP), SPO_2_, glucose, hemoglobin, white blood cell (WBC) count, platelet, bicarbonate, creatinine, sodium, potassium). Comorbidities (infection, myocardial infarct, congestive heart failure, peripheral vascular disease, cerebrovascular, chronic pulmonary disease, hypertension, and diabetes) were also recorded and the comorbidity disease index was calculated. AKI stage which was defined by KDIGO criteria was needed. Treatments (the use of mechanic ventilation and norepinephrine on day 1, surgery during the hospitalization) and disease severity score [simplified acute physiology score (SOFA), along with simplified acute physiology score (SAPS) II] were also required. The worst values of vital signs and laboratory tests on the first day were taken.

### Primary Outcome and Secondary Outcomes

The primary outcome of the study was in-hospital mortality. Secondary outcomes included the number of ICU-free days and norepinephrine-free days at 28 days after ICU admission, total fluid input, and fluid balance during the first and second day.

### Statistical Analysis

Descriptive analysis was performed for all participants. Categorical variables were expressed as numbers and percentages. Continuous variables were expressed as mean and standard deviation (SD) for normal distributions or median and interquartile range for skewed distributions. We used the chi-square test, *T*-test, and Kruskal-Wallis test for the comparison of categorical, normally distributed, and non-normally distributed continuous variables, respectively. We applied the Kaplan–Meier and log-rank analyses to determine survival curves.

A time–dose–response association between CVP wait time and in-hospital mortality was implemented to find the inflection point. The patients were divided into early and delayed CVP groups based on the inflection point.

Logistic regression, with propensity score matching (PSM), was used to estimate the probability that patients would measure CVP early and minimize the potential bias of allocation of CVP wait time and confounding. A 1:1 nearest neighbor matching algorithm was applied using a caliper width of 0.2. The variables mentioned above as covariates were selected to generate the propensity score. A standardized mean difference (SMD) was used to examine the degree of PSM. Less than 0.1 was considered an acceptable threshold.

In the PSM cohort, a 2-sided *t*-test was used to compare the second outcomes. The estimated propensity scores were used as weights. Pairwise algorithmic (PA) (16) and overlap weight (OW) (17) models were used to generate a weighted cohort. A doubly robust estimation (18) combines a multivariate Cox regression model with a propensity score model that was also used to estimate the independent associations in the full cohort.

All analyses were performed using the statistical software packages R 3.3.2 (http://www.R-project.org, The R Foundation) and Free Statistics software versions 1.4 (19). A two-tailed test was performed and *p* < 0.05 was considered statistically significant.

### Subgroup Analysis and Sensitivity Analysis

We conducted sensitivity studies only in the included patients with AKI within the first 48 h and repeated the study in MIMIC-III database. Several subgroup analyses were performed according to age, sex, norepinephrine used within day 1, high or low SOFA score groups, and surgical patients. We explored the potential for unmeasured confounding between CVP groups and in-hospital mortality by calculating *E*-values (20).

## Results

### Participants

**Figure 1:**
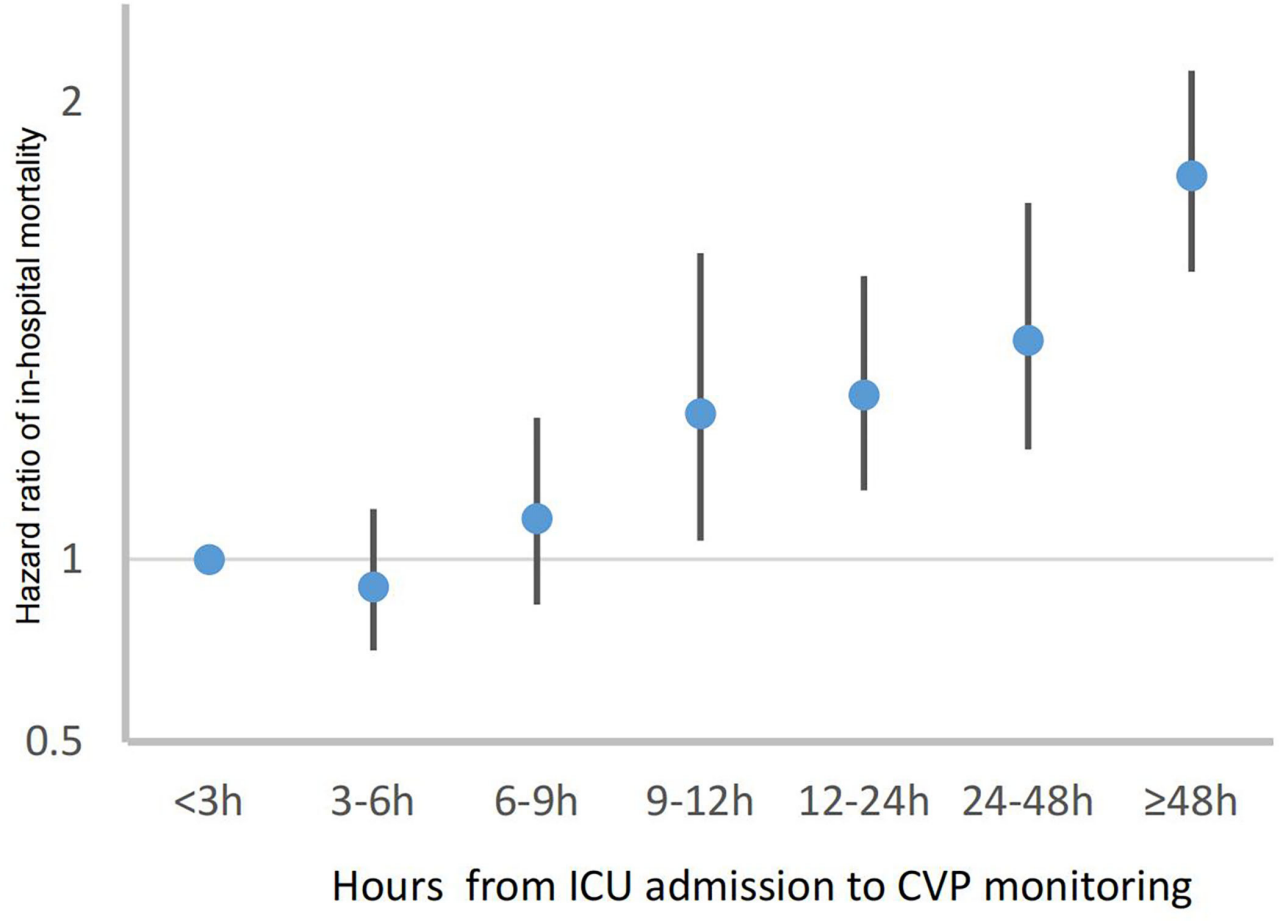
Time-dose-response relationship between CVP wait time and in-hospital mortality.

Of the 53,150 adult patients with first hospital and ICU admission in MIMIC-IV from 2008 to 2019, 29,336 patients with AKI were identified (Flowchart in Supplementary Figure 1). A time–doseresponse relationship between CVP wait time and in-hospital mortality was found in Cox proportional hazards regression model (Figure 1). Adjusted hazard ratios were graphically represented and an inflection point which was around 9 h existed when the risk of in-hospital mortality began to rise, irrespective of all confounders in Table 1. According to the premise of using inflection point as the grouping criteria, 8,071 (27.5%) patients received early CVP monitoring and 21,265 (72.5%) patients received delayed CVP monitoring beyond 9 h of ICU admission or did not receive CVP monitoring. In addition, 84 patients with CVP monitoring before ICU admission were included in the delayed CVP group.

**Table 1:** Baseline characteristics of participants.

**Covariate**		**Unmatched Patients**			**Propensity-Score–Matched Patients**		
	**All patients**	**CVP wait time**			**CVP wait time**		
		**Early (≤9 h)**	**Delayed (>9 h)**	**SMD**	**Early (≤9 h)**	**Delayed (>9 h)**	**SMD**
*N*	29,336	8,071	21,265		5,392	5,392	
Age (years), mean (SD)	67.7 ± 15.8	68.2 ± 12.8	67.5 ± 16.8	0.050	67.4 ± 13.3	67.1 ± 16.7	0.016
Male, sex, no. (%)	16,866 (57.5)	5,325 (66.0)	11,541 (54.3)	0.240	3,373 (62.6)	3,332 (61.8)	0.016
Admission year, no. (%)
2008–2010	9,427 (32.1)	2,517 (31.2)	6,910 (32.5)	0.045	1,720 (31.9)	1,726 (32.0)	0.009
2011–2013	7,048 (24.0)	2,023 (25.1)	5,025 (23.6)		1,341 (24.9)	1,355 (25.1)	
2014–2016	6,934 (23.6)	1,949 (24.1)	4,985 (23.4)		1,311 (24.3)	1,308 (24.3)	
2017–2019	5,927 (20.2)	1,582 (19.6)	4,345 (20.4)		1,020 (18.9)	1,003 (18.6)	
AKI stage, no. (%)							
1	7,846 (26.7)	2,440 (30.2)	5,406 (25.4)	0.172	1,451 (26.9)	1,410 (26.1)	0.032
2	14,614 (49.8)	4,131 (51.2)	10,483 (49.3)		2,717 (50.4)	2,687 (49.8)	
3	6,876 (23.4)	1,500 (18.6)	5,376 (25.3)		1,224 (22.7)	1,295 (24.0)	
Vital Signs, mean (SD)
Heart rate (bpm)	85.3 ± 15.9	84.3 ± 13.0	85.7 ± 16.8	0.097	85.2 ± 14.0	85.4 ± 16.5	0.015
MAP (mmHg)	77.4 ± 10.6	74.0 ± 7.3	78.6 ± 11.4	0.484	74.7 ± 7.8	74.5 ± 9.2	0.023
SpO_2_ (%)	96.9 ± 2.4	97.4 ± 2.3	96.7 ± 2.4	0.324	97.3 ± 2.5	97.2 ± 2.3	0.032
Laboratory tests
WBC (×10^9^/L), median (IQR)	13.3 (9.7, 18.0)	15.5 (12.0, 20.0)	12.4 (9.0, 17.0)	0.251	15.5 (11.9, 20.0)	13.6 (9.7, 18.8)	0.026
Hemoglobin (g/L), mean (SD)	10.0 ± 2.2	9.3 ± 1.8	10.3 ± 2.3	0.482	9.6 ± 1.8	9.6 ± 2.2	0.006
Platelet (×10^9^/L), median (IQR)	166.0 (117.0, 227.0)	134.0 (103.0, 174.0)	183.0 (129.0, 243.0)	0.537	143.5 (109.0, 189.0)	149.0 (100.0, 205.0)	0.010
Sodium (mmol/L), mean (SD)	139.7 ± 5.0	139.6 ± 3.8	139.8 ± 5.4	0.052	139.7 ± 4.1	139.7 ± 5.1	0.008
Potassium (mmol/L), mean (SD)	4.6 ± 0.8	4.7 ± 0.6	4.6 ± 0.9	0.128	4.7 ± 0.7	4.7 ± 0.9	<0.001
Bicarbonate (mmol/L), mean (SD)	21.5 ± 4.8	21.3 ± 3.8	21.5 ± 5.1	0.061	21.0 ± 4.2	21.0 ± 5.2	0.016
Creatinine (mg/dl), median (IQR)	1.1 (0.8, 1.7)	1.1 (0.8, 1.5)	1.1 (0.8, 1.8)	0.168	1.1 (0.8, 1.7)	1.2 (0.9, 1.8)	0.013
Glucose (mg/dl), median (IQR)	131.6 (115.0, 157.8)	130.3 (121.0, 145.5)	132.8 (111.0, 164.0)	0.016	130.8 (120.5, 148.7)	137.0 (114.5, 168.5)	<0.001
Comorbidity disease, *n* (%)
Infection	17,024 (58.0)	5,272 (65.3)	11,752 (55.3)	0.198	3,560 (66.0)	3,556 (65.9)	0.002
Myocardial infarct	5,567 (19.0)	1,958 (24.3)	3,609 (17.0)	0.178	1,211 (22.5)	1,188 (22.0)	0.01
Congestive heart failure	8,824 (30.1)	2,394 (29.7)	6,430 (30.2)	0.015	1,612 (29.9)	1,644 (30.5)	0.013
Peripheral vascular disease	3,765 (12.8)	1,340 (16.6)	2,425 (11.4)	0.149	804 (14.9)	806 (14.9)	0.001
Cerebrovascular disease	4,612 (15.7)	778 (9.6)	3,834 (18.0)	0.244	590 (10.9)	597 (11.1)	0.004
Chronic pulmonary disease	7,473 (25.5)	1,950 (24.2)	5,523 (26.0)	0.043	1,343 (24.9)	1,363 (25.3)	0.009
Hypertension	13,345 (45.5)	4,249 (52.6)	9,096 (42.8)	0.195	2,595 (48.1)	2,571 (47.7)	0.009
Diabetes	9,192 (31.3)	2,795 (34.6)	6,397 (30.1)	0.095	1,758 (32.6)	1,723 (32.0)	0.014
Severity of illness, median (IQR)
SAPS II score	37.0 (29.0, 47.0)	39.0 (31.0, 49.0)	37.0 (29.0, 46.0)	0.195	38.0 (31.0, 49.0)	40.0 (31.0, 50.0)	0.026
SOFA score	5.0 (3.0, 8.0)	6.0 (4.0, 9.0)	5.0 (3.0, 7.0)	0.393	6.0 (4.0, 9.0)	6.0 (4.0, 10.0)	0.036
Comorbidity index	6.0 (4.0, 8.0)	5.0 (4.0, 7.0)	6.0 (4.0, 8.0)	0.176	5.0 (4.0, 7.0)	6.0 (4.0, 7.0)	0.01
Interventions, *n* (%)
Mechanical ventilation (day 1)	13,113 (44.7)	5,500 (68.1)	7,613 (35.8)	0.678	3,205 (59.4)	3,268 (60.6)	0.024
Norepinephrine (day 1)	6,969 (23.8)	2,412 (29.9)	4,557 (21.4)	0.193	1,732 (32.1)	1,832 (34.0)	0.039
Surgery	9,718 (33.1)	4,847 (60.1)	4,871 (22.9)	0.811	2,425 (45.0)	2,401 (44.5)	0.009

### Baseline Characteristics

The baseline characteristics of all participants are listed in Table 1. The age of all participants was 67.7 ± 15.8, and 57.5% (16,866) were male. Patients in the early CVP group were older, had more serious conditions (higher levels of WBC, SOFA score, and SAPS II score, as well as higher rates of infection, myocardial infarct, peripheral vascular disease, and hypertension), and required more life support (higher use of mechanic ventilation and norepinephrine), while the incidence of cerebrovascular disease and comorbidity index was higher in the delayed CVP group. Patients in the early CVP group were more likely to be in stage 1 of AKI. A higher stage (stage 3) was more commonly seen among patients in the delayed CVP group (Table 1). The baseline characteristics of the two groups after PSM are balanced in Table 1, Supplementary Figure 2.

### Primary Outcome

The overall in-hospital mortality was 14.5% (4,260/29,336). In-hospital mortality of patients in the early CVP group and delayed CVP group was 9.4% (755/8,071) and 16.5% (3,505/21,265), respectively. Kaplan–Meier curve showed that an early measurement of CVP had lower mortality by day 30 (Log-rank test: *p* < 0.0001, Supplementary Figure 2).

**Figure 2:**
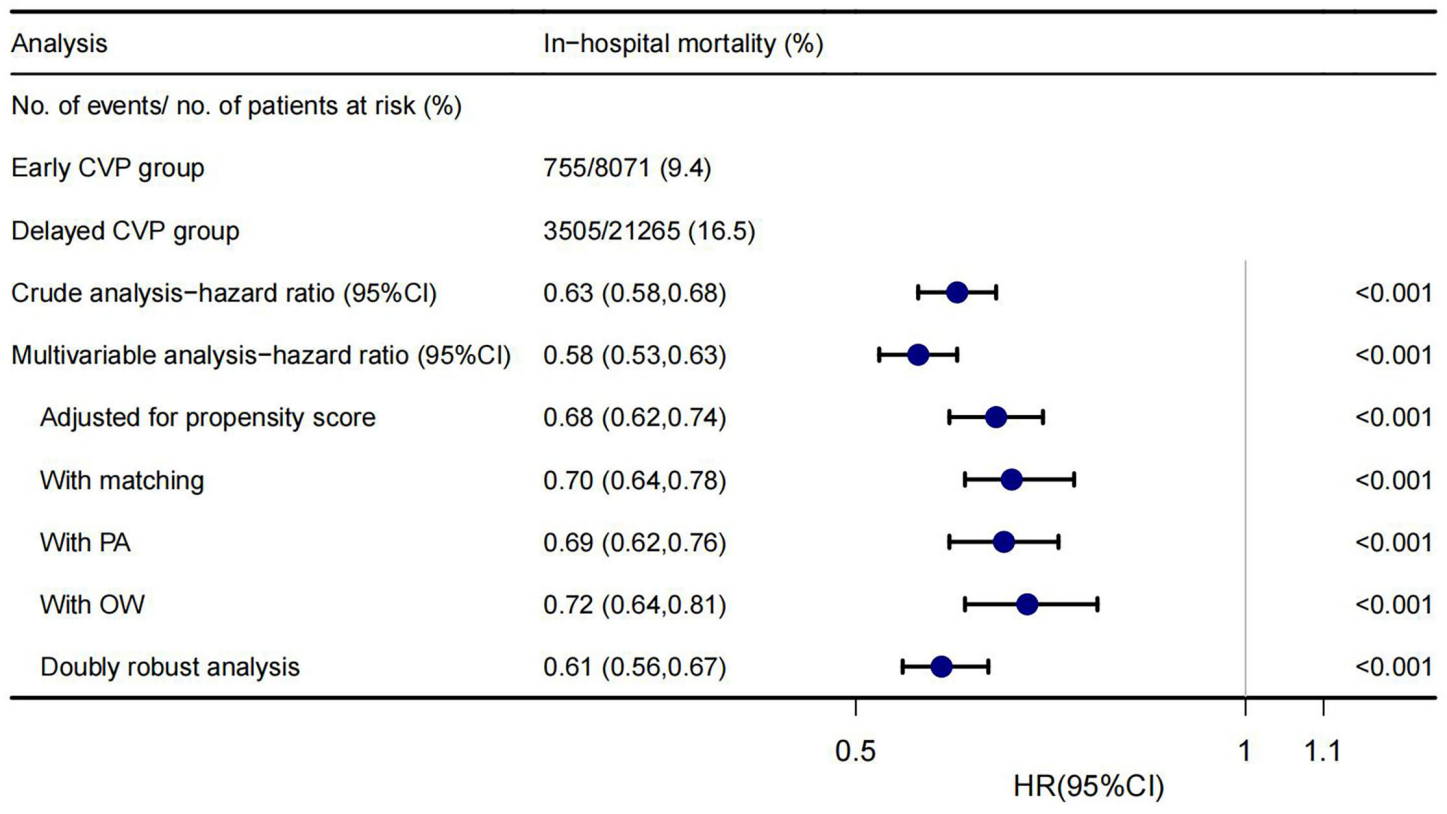
Forest plot shows HRs of in-hospital mortality in early CVP group using a variety of models.

A significantly lower in-hospital mortality in the early CVP group was found in univariate, multivariate Cox regression analyses, PSM, adjusted for propensity score, PA, OW, and doubly robust estimate. The HRs were 0.58–0.72, all *p* < 0.001 (Figure 2). The *E*-value of this cohort was 2.12–2.84.

### Secondary Outcome Analysis With PSM Cohorts

After balancing the confounding using PSM, the early CVP group had better survival outcomes, with a lower 28- and 90-day mortality than the delayed CVP group (10.9% vs. 17.8%, *p* < 0.001; 10.9% vs. 17.8%, *p* < 0.001; respectively, Table 2). Moreover, the number of ICU-free days and norepinephrine-free days was also higher in the early CVP group (21.2 ± 9.1 vs. 18.3 ± 10.6, *p* < 0.001; 20.8 ± 11.5 vs. 18.7 ± 12.6, *p* < 0.001; respectively, Table 2).

**Table 2:** Secondary outcome analysis after matching.

**Secondary outcomse**	**Propensity-Score–Matched Patients**		
	**CVP wait time**		***P* value**
	**Early (≤9 h) (*n* = 5,392)**	**Delayed (>9 h) (*n* = 5,392)**	
28-day mortality, *n* (%)	588 (10.9)	959 (17.8)	<0.001
90-day mortality, *n* (%)	649 (12)	1,061 (19.7)	<0.001
ICU free day, Mean (SD)	21.2 ± 9.1	18.3 ± 10.6	<0.001
Norepinephrine free day, Mean (SD)	20.8 ± 11.5	18.7 ± 12.6	<0.001
Input day 1 (L), Median (IQR)	3.73 (2.63, 5.18)	2.98 (1.66, 4.70)	<0.001
Input day 2 (L), Median (IQR)	0.96 (0.35, 0.20)	1.82 (0.79, 3.08)	<0.001
Fluid balance day 1 (L), Median (IQR)	2.16 (0.96, 3.72)	1.53 (0.21, 3.42)	<0.001
Fluid balance day 2 (L), Median (IQR)	−0.33 (−1.25, 0.60)	0.35 (−0.65, 1.68)	<0.001

More fluids were administered to the early CVP monitoring group on day 1 (3.73 vs. 2.98 L, *p* < 0.001, Table 2) and so did fluid balance (2.16 vs. 1.53 L, *p* < 0.001, Table 2). However, the delay group lagged by day 2 (1.82 L vs. 0.96 L, *p* < 0.001; 0.35 vs. −0.33 L, *p* < 0.001; respectively, Table 2).

### Sensitivity Analysis and Subgroup Analysis

**Figure 3:**
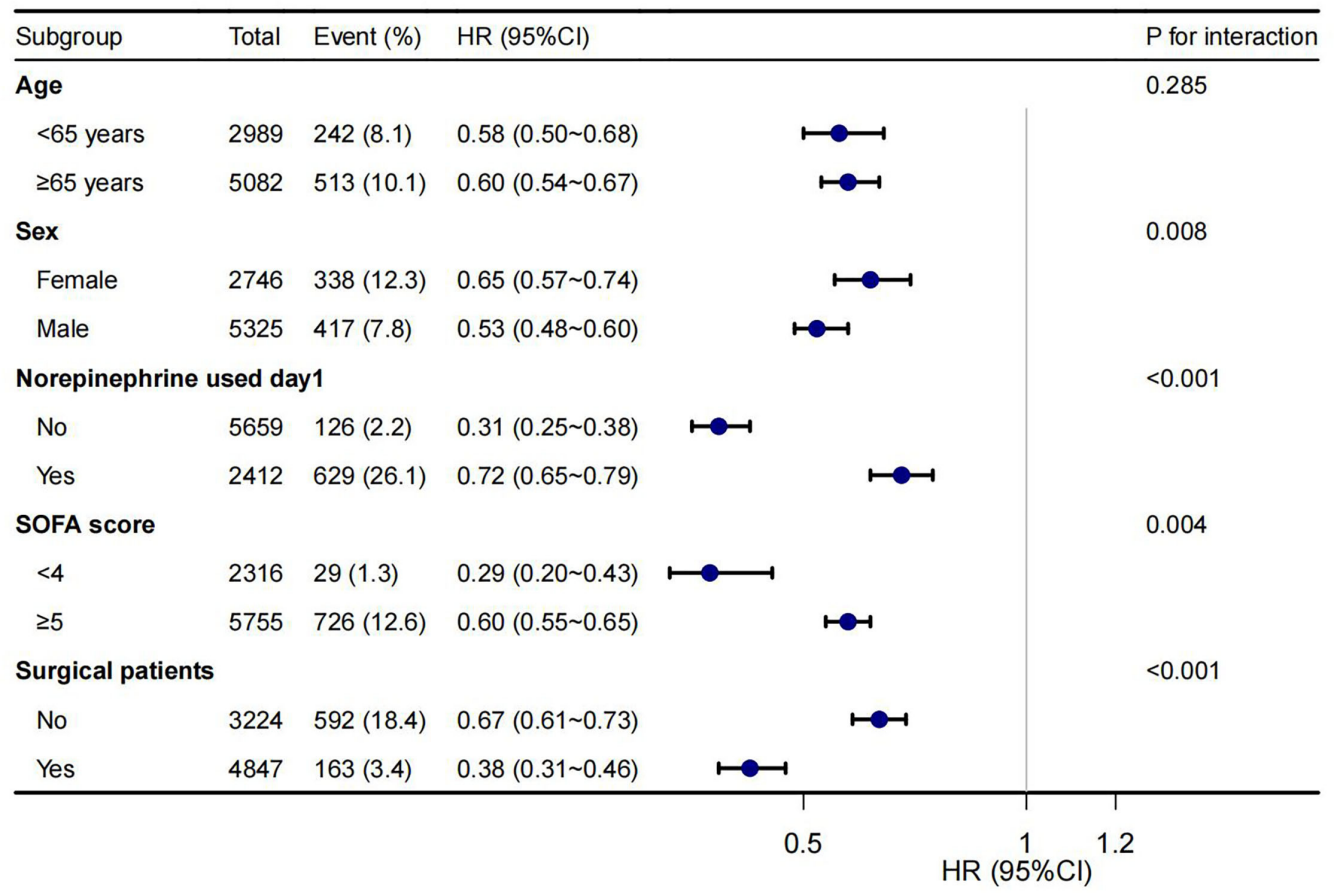
Forest plot shows HRs of in-hospital mortality in early CVP group in subgroup analyses.

A sensitivity study using the multivariate Cox regression model was performed only in patients with AKI within the first 48 h of ICU admission, and the association between early CVP measurement and lower in-hospital mortality was similarly observed (HR = 0.63, 95%CI 0.57–0.69, *p* < 0.001). In addition, we used a MIMIC-III cohort for further verification and found similar results (Supplementary Figure 3). Subgroup analysis showed that the relationship remained robust and reliable (Figure 3). However, some interactions were found in sex, norepinephrine used on day 1, SOFA score, and surgical patients.

## Discussion

Our study demonstrated that an increased CVP wait time beyond 9 h after ICU admission was associated with a higher risk of in-hospital mortality in patients with AKI, suggesting the necessity of early CVP monitoring. This result remained robust in the comparisons after PSM, PA, OW, doubly robust estimate, and subgroup analysis. HRs for *E*-value analysis were 2.12–2.84, indicating that unmeasured confounding could negate the observed effect. Furthermore, our findings were also suggestive of a possible beneficial role for early CVP monitoring in shortening the length of ICU stays and days of norepinephrine use, as well as fluid management.

Central venous pressure monitoring has been widely used since 1956 to guide fluid therapy in unstable patients (21). Nonetheless, the use of CVP monitoring in critical care patients has been questioned during the past decade (10). CVP is criticized for influencing many factors, such as thoracic, pericardial, and abdominal pressures (5). However, increasing numbers of studies recognize that CVP may be an indicator of outcomes in ICU. Long et al. (22) reported that elevated CVP had worse outcomes in patients with mechanical ventilation. Li et al. (23) also found elevated CVP levels correlated with poor outcomes and prolonged treatment in MIMIC-III database. In the VASST study, Boyd et al. (24) found that fluid overload and increased CVP (>12 mmHg) caused an increase in mortality in critically ill patients. A recent meta-analysis summarized in a previous work showed that elevated CVP was associated with an increased risk of mortality and AKI in critically ill adult patients admitted into the ICU (25). Rather than only focusing on the ambiguous values of CVP, our study tried to understand CVP measurement influence clinicians' decisions.

Chen et al. (26) found that CVP measurement within 24 h was associated with decreased risk-adjusted 28-day mortality among patients with sepsis. Our study extended a similar conclusion to patients with AKI. Rather than arbitrarily dividing patients into two groups, the division of early and delayed CVP groups was based on time–dose–response relationship between CVP wait time and adjusted in-hospital mortality. Compared with patients who did not complete CVP monitoring or had CVP monitoring later, adjusted in-hospital mortality was lower in 0–9 h group. The exact time-to-CVP monitoring data were used to empirically define a threshold for increased risk of death in our study.

Gao et al. (27) pointed out that ICU admissions after surgeries were more likely to have central venous catheters than those from the non-surgical patients in the ICU. In ICU, non-surgical patients also had a worse prognosis than surgical patients (28, 29). Therefore, whether patients have surgery or not might be a very important confounder in the studies about CVP (28). We adjusted this confounder and did a further subgroup analysis. The results showed early CVP monitoring was associated with lower in-hospital mortality in both surgical and non-surgical patients. However, the effect of early CVP monitoring in surgical patients was significantly better than in non-surgical patients, consistent with Gao's expectations (27). In addition, we also found early CVP monitoring had a more powerful effect on outcome in male patients, who used norepinephrine on day 1, and who obtained less SOFA score. These associations were worthy of further investigation.

Consistent with our clinical experiences, the severity of illness of the early CVP monitoring group was worse than the delayed CVP monitoring group. Even so, we still found significantly lower adjusted in-hospital mortality in patients with early CVP monitoring. This relationship was also confirmed in the MIMIC-III database.

The reason for early CVP measurement being associated with lower mortality in patients with AKI is still unclear. There were many causes of AKI, and prerenal factors were only one of them. The improvement of prognosis in patients with AKI might be multifaceted. Even so, it was known that CVP was affected by cardiac function, circulating blood volume, and vascular tension, and early monitoring of CVP might be conducive to early etiology searches (such as abdominal hypertension and right heart failure, etc.) and early intervention. In addition, one of the most important roles of early CVP monitoring was fluid management.

The value of CVP has been criticized as a poor predictor of hemodynamic responsiveness since it is influenced by many factors (5). However, it is argued that CVP monitoring provides important physiologic information for the evaluation of hemodynamic instability (5). In our findings, fluid input increased first and then decreased in the early CVP monitoring group during the first 2 days after admission to the ICU, which suggests that early fluid resuscitation, followed by a negative fluid balance possibly improved prognosis in patients with AKI (30, 31). It was mediately reflected that CVP might provide important information on fluid load to help clinicians maintain a better fluid administration. However, it was important to note that increased volume of fluid input in early fluid resuscitation would also lead to fluid overload in patients, which might also have certain negative effects. Defectively, our study only studied the CVP time of the first measurement, so it was particularly important to continuously monitor CVP during the subsequent course of the disease for early detection of disease changes.

## Limitations

This study has several noteworthy limitations. First, some residual confounders may potentially exist, as with all retrospective analyses. We adjusted for possible confounders and minimized the influence of factors that may lead to outcome bias through the PSM, PA, OW, and doubly robust estimate. Second, as the study population only contains patients with AKI, it may not be generalizable to patients without AKI. Third, CVP grouping might induce some bias. We grouped according to time–dose–response effects rather than arbitrarily grouping the patients. Fourth, patients with CVP monitoring before ICU admission were divided into a delayed CVP group. Meanwhile, immortal time bias might exist in the delay CVP group. However, the two conditions above seemed to be misclassification resulting in some bias, led to an underestimation of the association between early central venous pressure monitoring and in-hospital mortality.

## Conclusions

Among adults with AKI in ICU, an increased CVP wait time was associated with a greater risk of in-hospital mortality. In addition, early CVP monitoring perhaps contributed to shortening the length of ICU stays and days of norepinephrine use, as well as better fluid management.

## Data Availability Statement

The raw data supporting the conclusions of this article will be made available by the authors, without undue reservation.

## Ethics Statement

The studies involving human participants were reviewed and approved by Massachusetts Institute of Technology and Beth Israel Deaconess Medical Center. Written informed consent for participation was not required for this study in accordance with the national legislation and the institutional requirements.

## Author Contributions

QY and WeixC conducted data analysis and wrote the manuscript. YW and JZ modified the manuscript and interpreted the analysis. JC and SY conducted the data collection. XC and WeiyC conducted data analysis and reviewed the manuscript. XX conducted data collection and data interpretation. DW and ZZ designed the study and conducted data analysis and reviewed the manuscript. All authors contributed to the article and approved the submitted version.

## Conflict of Interest

The authors declare that the research was conducted in the absence of any commercial or financial relationships that could be construed as a potential conflict of interest.

## Publisher's Note

All claims expressed in this article are solely those of the authors and do not necessarily represent those of their affiliated organizations, or those of the publisher, the editors and the reviewers. Any product that may be evaluated in this article, or claim that may be made by its manufacturer, is not guaranteed or endorsed by the publisher.
