# Supplementary figures and images for: Association Between Wait Time of Central Venous Pressure Measurement and Outcomes in Critical Patients With Acute Kidney Injury: A Retrospective Cohort Study
Source: Front Public Health. 2022 Aug 9;10:893683. doi: 10.3389/fpubh.2022.893683 (PMC9395608; doi:10.3389/fpubh.2022.893683)

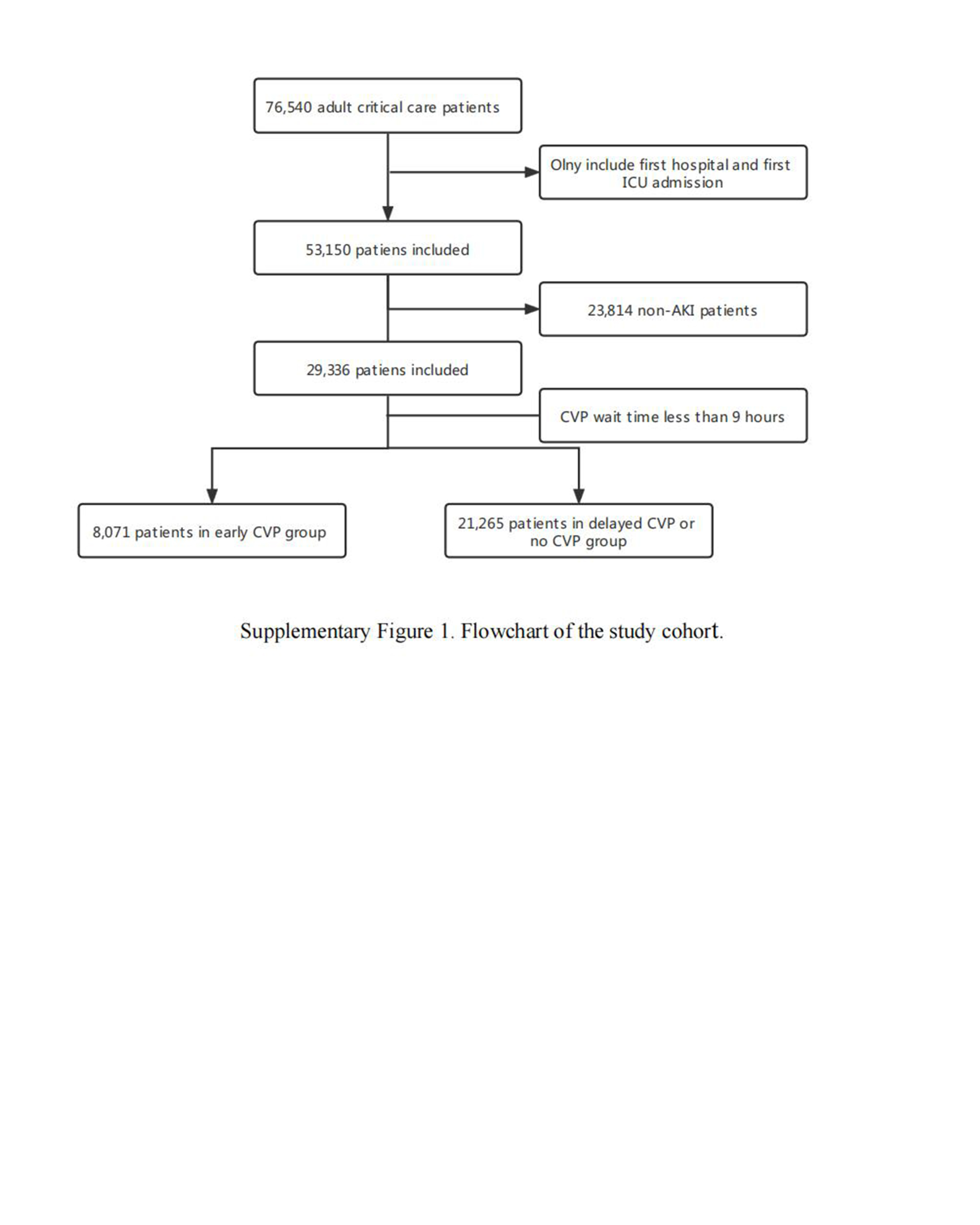

Supplement: Supplementary file 1 [file Image_1.JPEG]

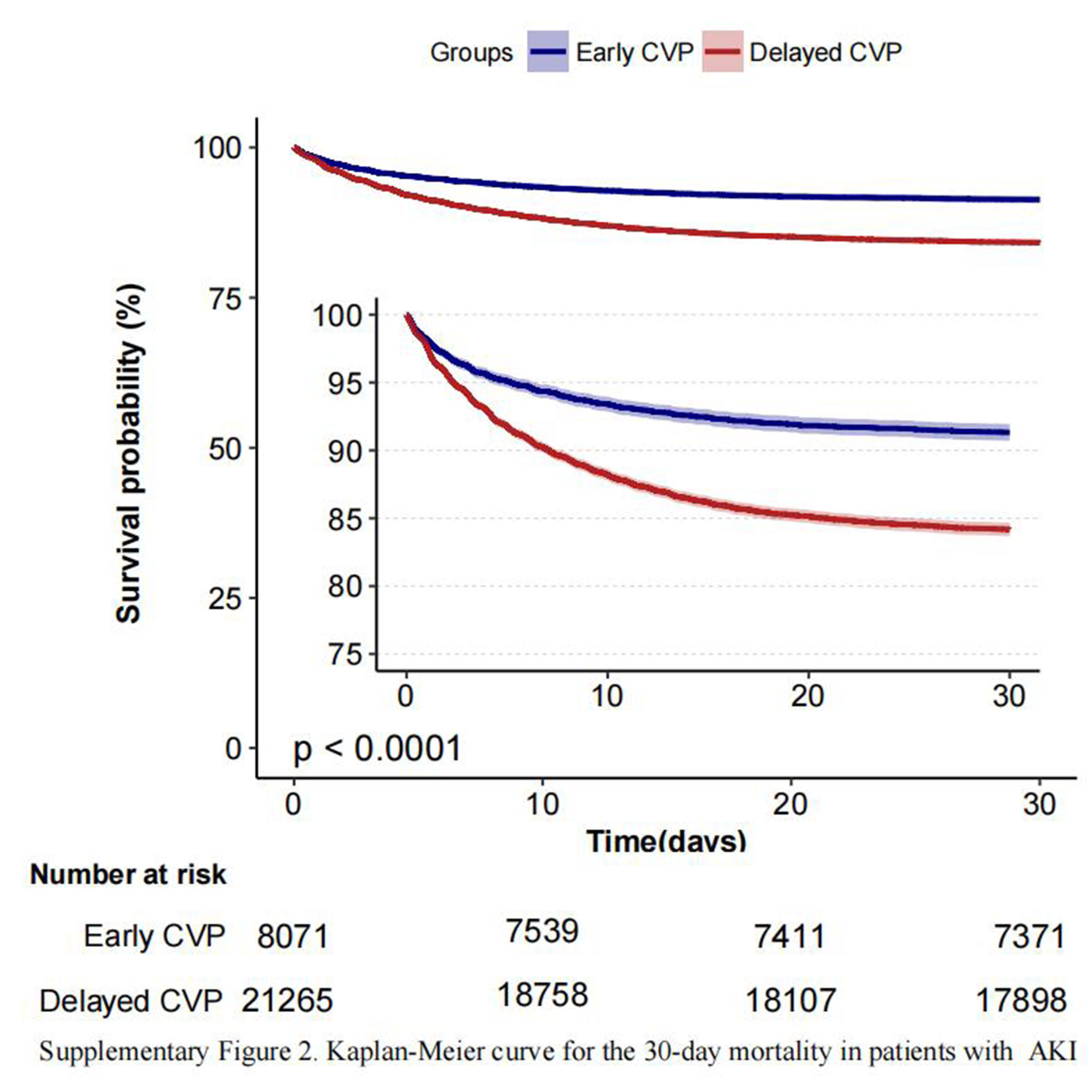

Supplement: Supplementary file 2 [file Image_2.JPEG]

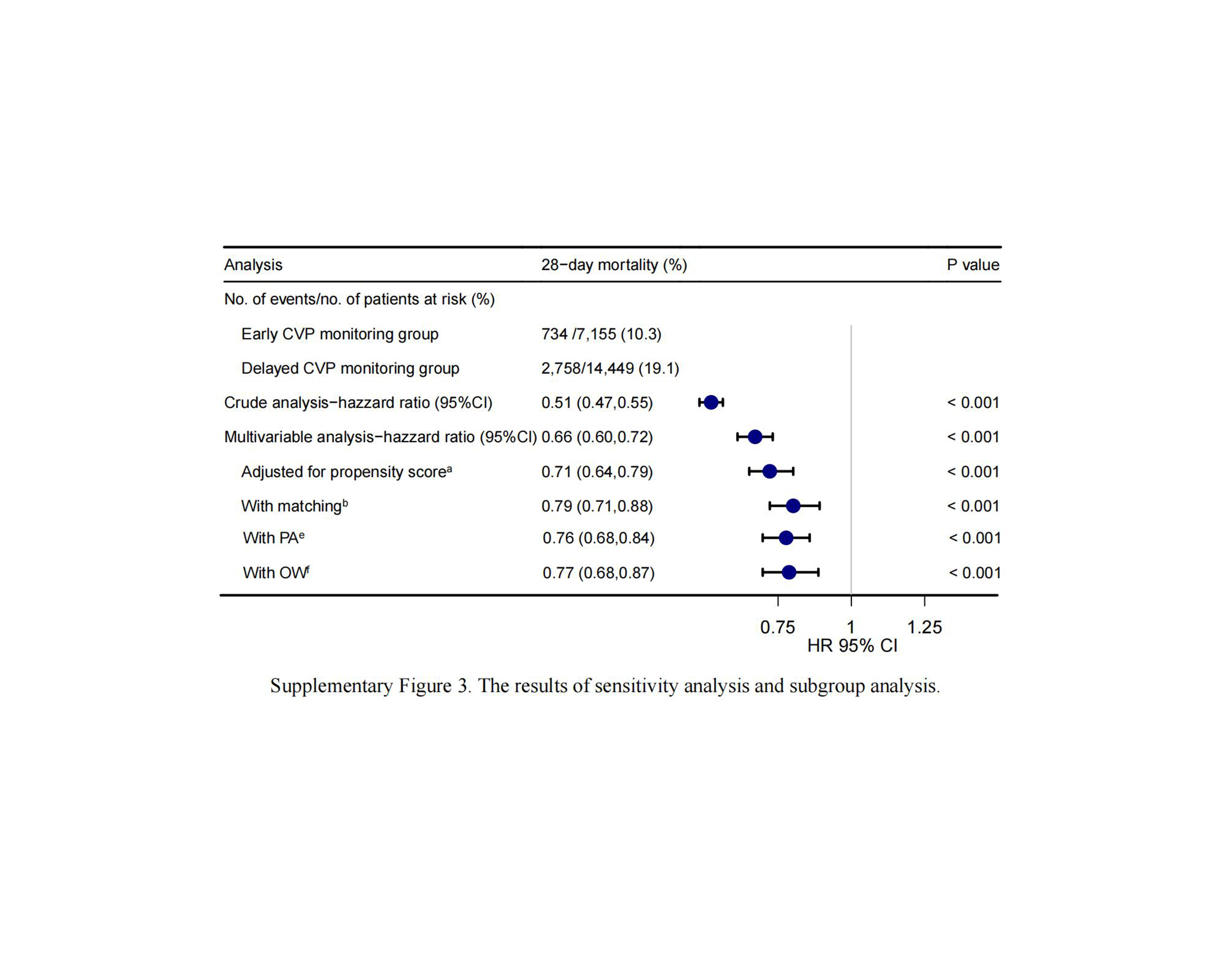

Supplement: Supplementary file 3 [file Image_3.JPEG]
